# The association between four scoring systems and 30-day mortality among intensive care patients with sepsis: a cohort study

**DOI:** 10.1038/s41598-021-90806-2

**Published:** 2021-05-27

**Authors:** Tianyang Hu, Huajie Lv, Youfan Jiang

**Affiliations:** 1 Department of Cardiology, The Second Affiliated Hospital, Chongqing Medical University, Chongqing, China; 2 Department of Infectious Diseases, Southwest Hospital, Third Military Medical University (Army Medical University), Chongqing, China; 3 Department of Respiration, The Second Affiliated Hospital, Chongqing Medical University, 74 Linjiang Road, Yuzhong District, Chongqing, 400010 China

**Keywords:** Medical research, Experimental models of disease

## Abstract

Several commonly used scoring systems (SOFA, SAPS II, LODS, and SIRS) are currently lacking large sample data to confirm the predictive value of 30-day mortality from sepsis, and their clinical net benefits of predicting mortality are still inconclusive. The baseline data, LODS score, SAPS II score, SIRS score, SOFA score, and 30-day prognosis of patients who met the diagnostic criteria of sepsis were retrieved from the Medical Information Mart for Intensive Care III (MIMIC-III) intensive care unit (ICU) database. Receiver operating characteristic (ROC) curves and comparisons between the areas under the ROC curves (AUC) were conducted. Decision curve analysis (DCA) was performed to determine the net benefits between the four scoring systems and 30-day mortality of sepsis. For all cases in the cohort study, the AUC of LODS, SAPS II, SIRS, SOFA were 0.733, 0.787, 0.597, and 0.688, respectively. The differences between the scoring systems were statistically significant (all *P*-values < 0.0001), and stratified analyses (the elderly and non-elderly) also showed the superiority of SAPS II among the four systems. According to the DCA, the net benefit ranges in descending order were SAPS II, LODS, SOFA, and SIRS. For stratified analyses of the elderly or non-elderly groups, the results also showed that SAPS II had the most net benefit. Among the four commonly used scoring systems, the SAPS II score has the highest predictive value for 30-day mortality from sepsis, which is better than LODS, SIRS, and SOFA. The results of the DCA curves show that using the SAPS II score to predict the 30-day mortality of intensive care patients with sepsis to guide clinical applications may obtain the highest net benefit.

## Introduction

The third international consensus definitions for sepsis and septic shock (Sepsis-3) were released in 2016^1^. Sepsis is defined as life-threatening organ dysfunction caused by a dysregulated host response to infection. Septic shock is defined as a subset of sepsis and can be identified with a clinical construct of sepsis with persisting hypotension requiring vasopressors to maintain mean arterial pressure (MAP) ≥ 65 mmHg and having a serum lactate level > 2 mmol/L despite adequate volume resuscitation. Sepsis has become an important public health issue worldwide, and its overall mortality rate is about 30%, especially in the intensive care unit (ICU)^1,2^. Sepsis-3 emphasized the strong association between infection and organ failure^1^, thus, a scoring system for organ dysfunction may be of great benefit to early-recognition of sepsis, especially for patients admitted to the ICU.

As the most commonly used severe disease scoring system in clinical practice, Sequential Organ Failure Assessment (SOFA) score is listed as the diagnostic criteria for sepsis (Sepsis-3)^1^, and has been proven to be effective in evaluating the prognosis of patients with sepsis^3^. Simplified Acute Physiology Score (SAPS II) was proposed in 1993^4^, and a study showed that patients with an admission diagnosis of sepsis/septic shock had the highest values of SAPS II^5^. The Logistic Organ Dysfunction System (LODS) provides an objective tool for assessing severity levels for organ dysfunction in the ICU^6^, each variable included in the LODS score is screened and weighted by Logistic regression. However, a previous study showed that the difference between LODS score and SOFA score in predicting the prognosis of sepsis was not statistically significant^7^. The task force of Sepsis-3 replaced the systemic inflammatory response syndrome (SIRS) criteria with SOFA score due to lacking discriminant validity and convergent validity^1^. Relative simplification is the advantage of SIRS criteria, and its net benefit of predicting mortality is still inconclusive. This study intends to explore the association between the four scoring systems (SOFA, SAPS II, LODS, and SIRS) and 30-day mortality of sepsis based on the MIMIC-III (Medical Information Mart for Intensive Care III) ICU database, to determine which scoring system could better predict 30-day mortality of sepsis and septic shock from the beginning of ICU admission. Considering that elderly patients with sepsis often present with atypical, nonspecific symptoms, and have greater mortality risks due to delay in time to diagnosis^8,9^, we will conduct a stratified analysis of elderly and non-elderly patients to determine whether age affects the efficacy of the scoring systems. In particular, we expect to discuss the net benefits between the scoring systems and 30-day mortality of sepsis through the decision curve analysis (DCA), a suitable method for evaluating alternative diagnostic and prognostic strategies^10^.

## Methods

### Database

MIMIC-III is a large, freely-available database comprised of over forty thousand patients admitted to the Beth Israel Deaconess Medical Center (BIDMC) between 2001 and 2012^11^. Any researcher who complies with the data use requirements is permitted to use the database. After passing the “Protecting Human Research Participants” exam on the website of the National Institutes of Health (NIH), an author (Tianyang Hu) was approved to extract data from this database (Record ID: 37474354). All patient-related information in the MIMIC-III database is anonymous and no informed consent is required.

### Study population

We followed the method of Johnson et al.^12^ to screen patients in the MIMIC-III ICU database from years 2008 to 2012 (the reason was that the group of admissions between 2008 and 2012 were easily identifiable in the database) that met the Sepsis-3 criteria, the core criteria for sepsis were extracted as suspected infection with associated organ dysfunction (SOFA greater than or equal to 2). All patients were required to have at least 24 h of ICU data. Finally, 5784 patients were identified as meeting the criteria, which was consistent with the results of Johnson et al.^12^. Meanwhile, we conducted a stratified analysis of the elderly (more than 65 years old) and non-elderly.

### Data extraction

Data were acquired from the MIMIC-III database (v1.4). PostgreSQL 10.7 and Navicat Premium 15.0 software were used to extract the basic characteristics (subject id, ICU stay id, age, gender), septic shock or not, coexisting comorbidities(coronary atherosclerotic heart disease, diabetes, hypertension, chronic pulmonary disease, and renal failure) of the patients that met the Sepsis-3 criteria from MIMIC-III database by SQL language (Structure query language), and extracted the relevant items to calculate the four scoring systems (SOFA, SAPS II, LODS, and SIRS) with the help of the MIMIC-III Concepts provided by Github community (https://github.com/MIT-LCP/mimic-code/tree/master/concepts).

### Statistical analysis

Continuous variables were assessed for normality using the Kolmogorov–Smirnov test. Continuous variables with a normal distribution were expressed as mean ± standard deviation (M ± SD), and the independent sample t test was used for the comparison; if the distribution was not normal, continuous variables were expressed as the median with interquartile range (IQR), and the Wilcoxon rank-sum test was used for comparison. Categorical variables were expressed as numbers and percentages, and compared using the Chi-square test. Multiple and binomial logistic regression analysis of the four scoring systems for 30-day mortality among intensive care patients with sepsis were conducted to adjust the results of the statistical analysis for potential confounding factors. Variables with a *P*-value of < 0.1 in univariate analysis were included in multivariate analysis. Z test was used to compare the predictive value of each scoring system by comparing the area under curves (AUC) of the receiver operating characteristic curves (ROC), and the larger the AUC, the better the predictive performance. All the analyses were conducted using SPSS software (v26.0; IBM, Armonk, NY), MedCalc Statistical Software (v19.6.1; MedCalc Software Ltd, Ostend, Belgium), and R software (version 4.0.3, CRAN). Among them, Z test was performed with Medcalc Statistical Software following the method of Delong et al.^13^; the DCA was performed with R software, mainly using the “rmda” package. A *P*-value < 0.05 was considered to be statistically significant.

## Results

### Baseline characteristics

A total of 5784 sepsis patients (elderly, n = 3138; non-elderly, n = 2646) were included in our study, in which, 1042 died and 4742 survived within 30 days. The age of the death group (71.66 ± 15.61) was higher than that of the survival group (64.17 ± 17.77), and the difference was statistically significant (*P* < 0.001). In addition, in the death group, the incidence of septic shock and coexisting comorbidities (chronic pulmonary disease and renal failure) was higher (*P* < 0.001, *P* = 0.004, and *P* = 0.006, respectively) and the four scoring systems (SOFA, SAPS II, LODS, and SIRS) scored higher (all *P* < 0.001). The gender and other coexisting comorbidities (coronary atherosclerotic heart disease, diabetes, and hypertension) show no significant difference between the two groups. The baseline data are summarized in Table 1.

**Table 1:** Demographic data of the study population.

Characteristics	Death group	Survival group	*P*
**N (sample size)**	1042	4742	
**Baseline variables**			
Age (median [IQR])	74.88 [60.93,83.99]	65.53 [52.65,78.47]	< 0.001
Gender (% Male)	558 (53.6)	2663 (56.2)	0.125
**Septic shock**	260 (24.9)	480 (10.1)	< 0.001
**Coexisting comorbidities**			
Coronary atherosclerotic heart disease	219 (21.0)	1117 (23.6)	0.078
Diabetes	288 (27.6)	1342 (28.3)	0.668
Hypertension	182 (17.5)	724 (15.3)	0.078
Chronic pulmonary disease	246 (23.6)	931 (19.6)	0.004
Renal failure	219 (21.0)	825 (17.4)	0.006
**Scoring systems**			
LODS (median [IQR])	7 [5,9]	4 [3, 6]	< 0.001
SAPS II (median [IQR])	51 [41, 63]	36 [28, 44]	< 0.001
SIRS (median [IQR])	3 [3, 4]	3 [2, 4]	< 0.001
SOFA (median [IQR])	7 [4, 10]	4 [3, 6]	< 0.001

### Comparison of ROC curves

**Figure 1:**
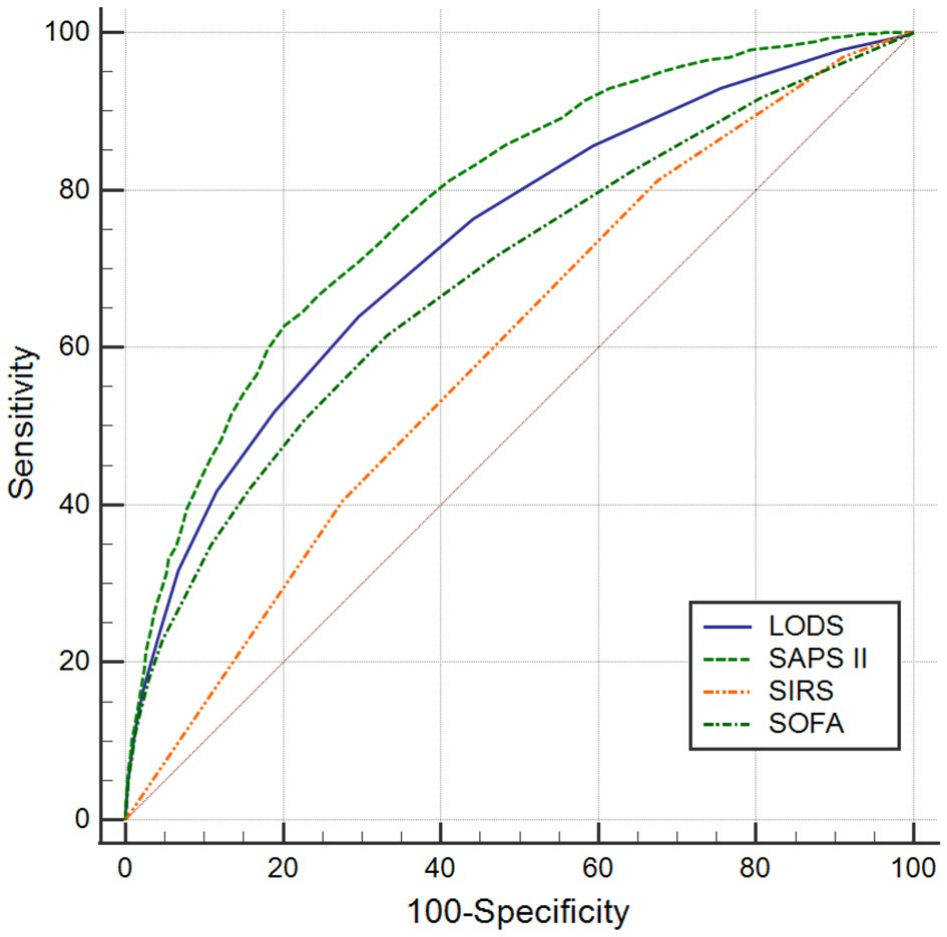
ROC curves of the four scoring systems for all cases in the cohort study.

ROC curves were performed to evaluate the predictive value of four scoring systems for 30-day mortality for all cases in the cohort study (Fig. 1). The AUC of LODS, SAPS II, SIRS, SOFA were 0.733, 0.787, 0.597, and 0.688, respectively. The AUC of four scoring systems were compared with each other, LODS versus SAPS II (Z = 8.810, *P* < 0.0001), LODS versus SIRS (Z = 12.736, *P* < 0.0001), LODS versus SOFA (Z = 6.602, *P* < 0.0001), SAPS II versus SIRS (Z = 18.055, *P* < 0.0001), SAPS II versus SOFA (Z = 12.566, *P* < 0.0001), SIRS versus SOFA (Z = 7.924, *P* < 0.0001). The cut-off value corresponding to the Youden’s index of each scoring system was selected as the diagnostic optimal cut-off value for predicting the 30-day mortality. SIRS criteria had the highest sensitivity of 81.2%, while the corresponding Youden’s index was the lowest, 0.137; the SAPS II score had the highest specificity at 79.8%, and its Youden’s index was also the highest, with a corresponding sensitivity of 62.8%. The remaining results are summarized in Table 2.

**Table 2:** Comparison of ROC curves for all cases in the cohort study.

Scoring system	AUC	95%CI	Optimal cut-off	Sensitivity	Specificity	Youden’s index
LODS	0.733	0.716–0.751	5.5	0.639	0.704	0.343
SAPS II	0.787	0.772–0.802	46.5	0.628	0.798	0.426
SIRS	0.597	0.578–0.615	2.5	0.812	0.325	0.137
SOFA	0.688	0.669–0.706	5.5	0.615	0.668	0.283

**Figure 2:**
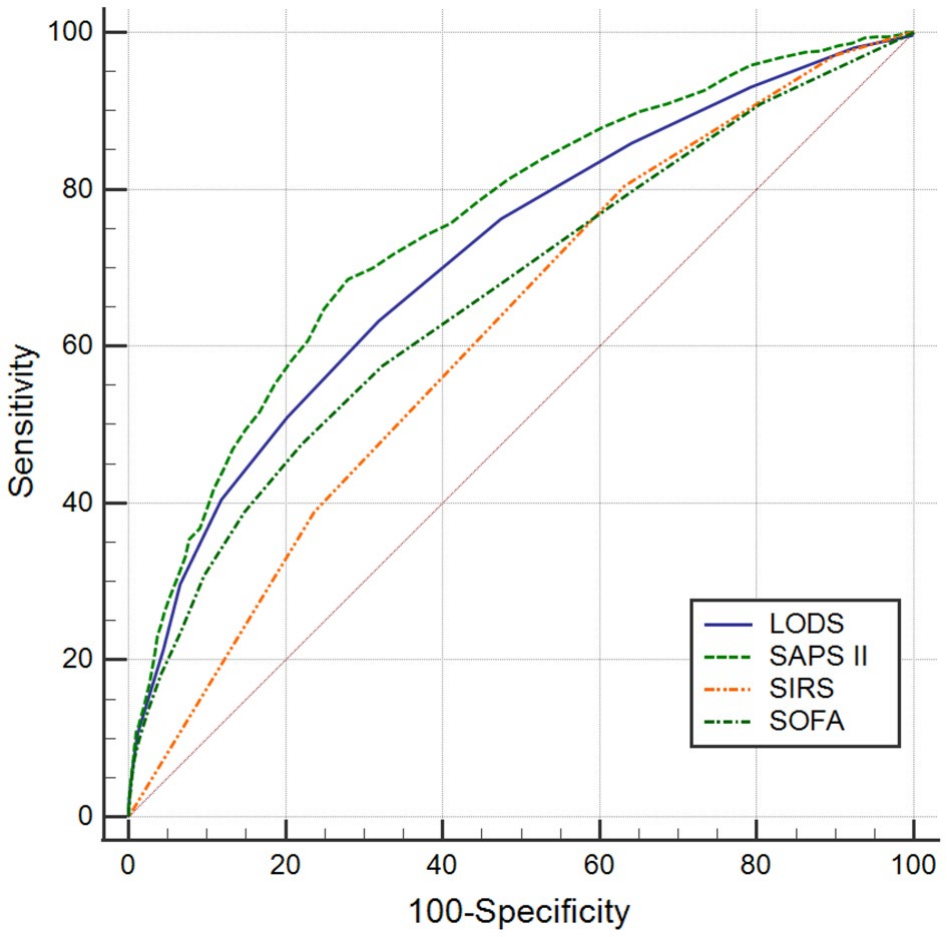
ROC curves of the four scoring systems for the elderly.

For the elderly (Fig. 2), the AUC of LODS, SAPS II, SIRS, SOFA were 0.715, 0.754, 0.619, and 0.665, respectively. The results of the AUC comparisons were as follows: LODS versus SAPS II (Z = 5.122, *P* < 0.0001), LODS versus SIRS (Z = 7.075, *P* < 0.0001), LODS versus SOFA (Z = 5.796, *P* < 0.0001), SAPS II versus SIRS (Z = 10.127, *P* < 0.0001), SAPS II versus SOFA (Z = 9.417, *P* < 0.0001), SIRS versus SOFA (Z = 3.280, *P* = 0.0010). Similarly, SIRS criteria had the highest sensitivity (80.4%), but the Youden’s index (0.173) was the smallest; the SAPS II score had the highest specificity (0.721) and Youden’s index (0.406). The results are summarized in Table 3.

**Table 3:** Comparison of ROC curves for the elderly.

Scoring system	AUC	95%CI	Optimal cut-off	Sensitivity	Specificity	Youden’s index
LODS	0.715	0.693–0.737	5.5	0.632	0.681	0.313
SAPS II	0.754	0.734–0.775	46.5	0.685	0.721	0.406
SIRS	0.619	0.596–0.641	2.5	0.804	0.369	0.173
SOFA	0.665	0.642–0.689	6.5	0.474	0.780	0.254

**Figure 3:**
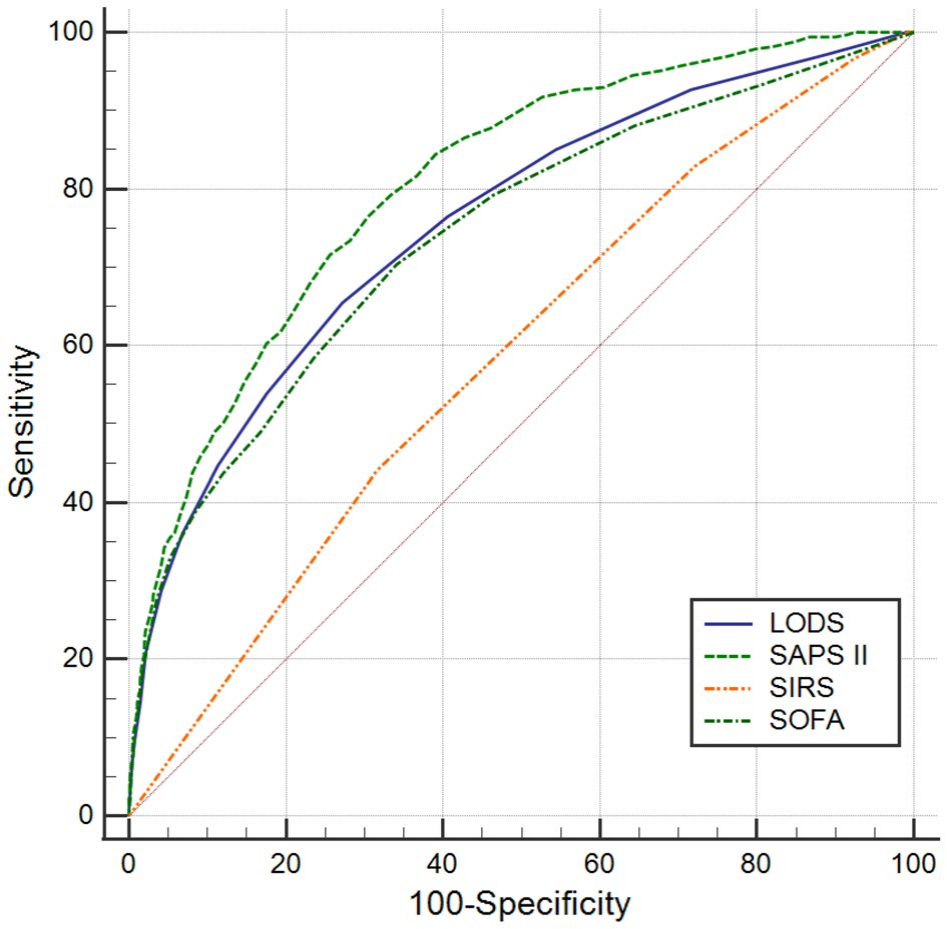
ROC curves of the four scoring systems for the non-elderly.

As to the non-elderly (Fig. 3), the AUC of LODS, SAPS II, SIRS, SOFA were 0.756, 0.808, 0.584, and 0.741, respectively. The results of the AUC comparisons were as follows: LODS versus SAPS II (Z = 5.342, *P* < 0.0001), LODS versus SIRS (Z = 9.539, *P* < 0.0001), LODS versus SOFA (Z = 1.230, *P* = 0.2186), SAPS II versus SIRS (Z = 12.604, *P* < 0.0001), SAPS II versus SOFA (Z = 5.365, *P* < 0.0001), SIRS versus SOFA (Z = 8.058, *P* = 0.0010). LODS had the highest specificity (72.8%) and SAPS II score had the highest sensitivity (76.5%), Youden’s index (0.461), and. The results are summarized in Table 4.

**Table 4:** Comparison of ROC curves for the non-elderly.

Scoring system	AUC	95%CI	Optimal cut-off	Sensitivity	Specificity	Youden’s index
LODS	0.756	0.727–0.785	5.5	0.654	0.728	0.382
SAPS II	0.808	0.783–0.832	36.5	0.765	0.696	0.461
SIRS	0.584	0.552–0.616	3.5	0.440	0.684	0.124
SOFA	0.741	0.710–0.771	5.5	0.703	0.659	0.362

### Comparison of decision curves

**Figure 4:**
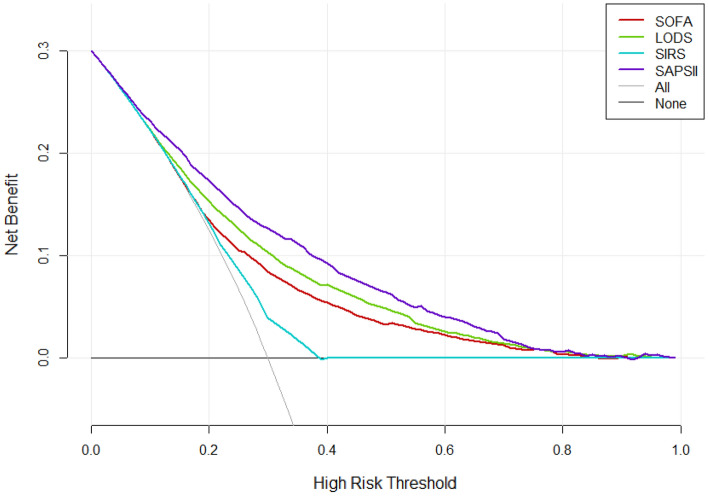
Decision curve analysis (DCA) of the four scoring systems for all cases in the cohort study. The net benefit curves for the four scoring systems are shown. X-axis indicates the threshold probability for clinical outcome and Y-axis indicates the net benefit. Solid red line = SOFA, solid green line = LODS, solid blue line = SIRS, solid purple line = SAPS II. The preferred scoring system is SAPS II, the net benefit of which was the largest among the four scoring systems.

**Figure 5:**
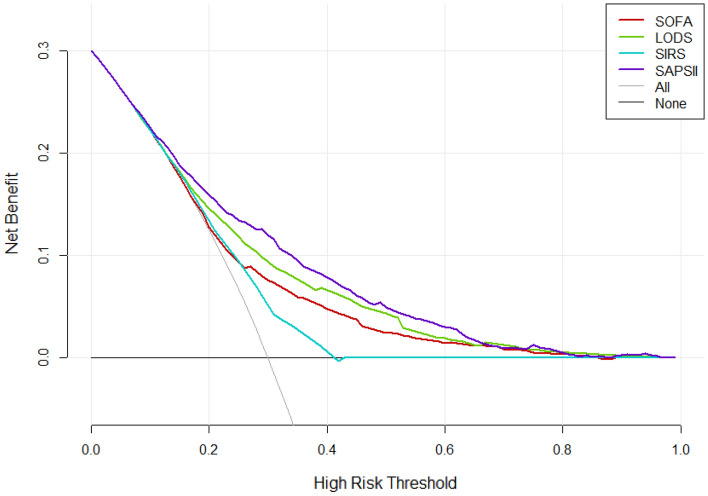
Decision curve analysis (DCA) of the four scoring systems for the elderly.

**Figure 6:**
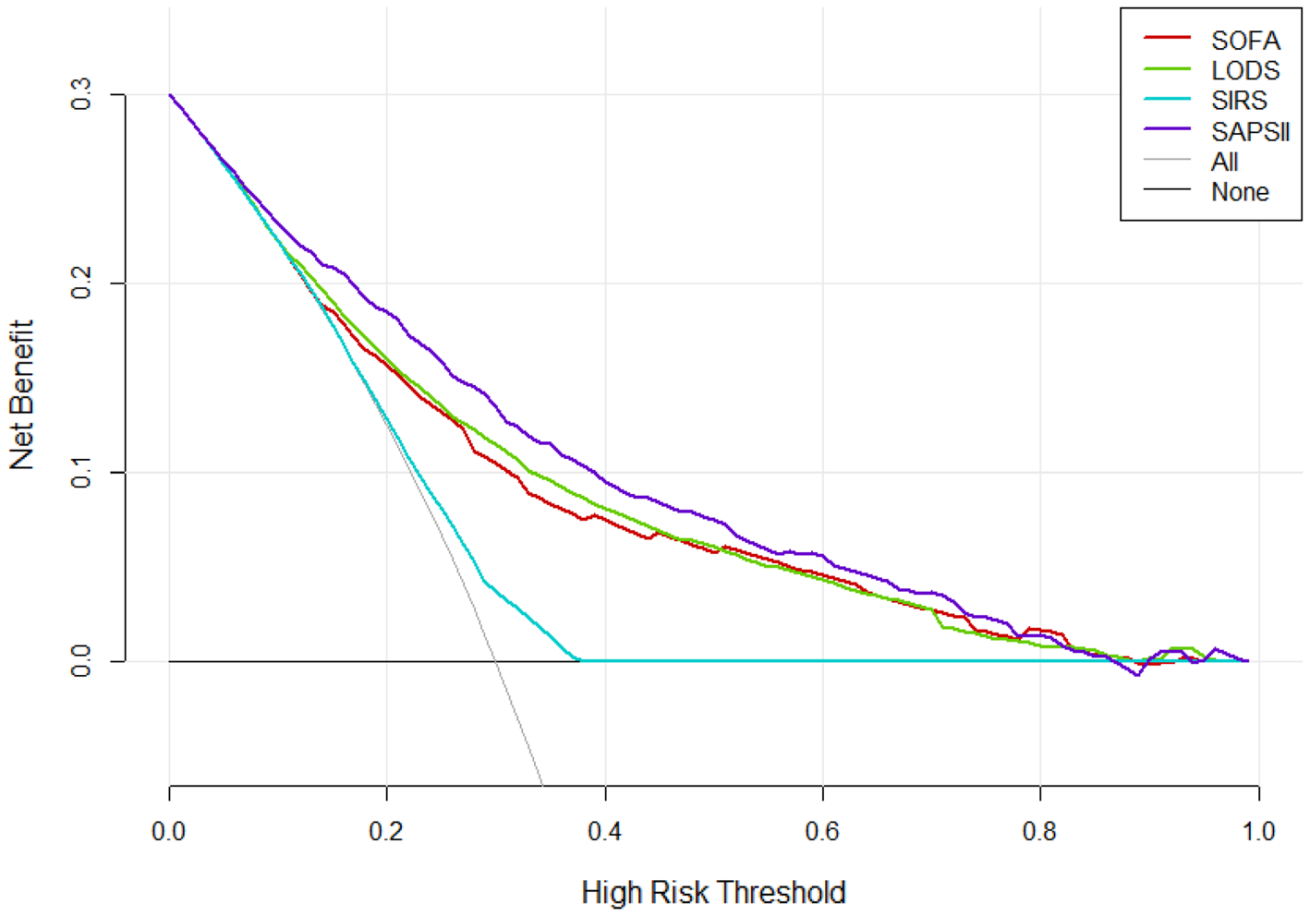
Decision curve analysis (DCA) of the four scoring systems for the non-elderly.

According to the DCA, the net benefit ranges in descending order were SAPS II, LODS, SOFA, and SIRS, which means SAPS II was optimal among the four scoring systems (Fig. 4). For stratified analyses of the elderly or non-elderly groups, the results also showed that SAPS II had the most net benefit (Figs. 5 and 6).

### Logistic regression analysis

Before the adjustment, the four scoring systems were all risk factors for 30-day mortality in intensive care patients with sepsis (all *P* < 0.001). Variables with a *P*-value of < 0.1 in univariate regression analysis (age, septic shock, coronary atherosclerotic heart disease, hypertension, chronic pulmonary disease, renal failure, LODS, SAPS II, SIRS, and SOFA) were recruited into the multivariate regression analysis, and the results showed that age, septic shock, coronary atherosclerotic heart disease, and chronic pulmonary disease were all independent risk factors for 30-day mortality; Of the four scoring systems, SAPS II and SIRS were independent risk factors for 30-day mortality (OR: 1.061, 95%: 1.051–1.071, *P* < 0.001; OR: 1.264, 95%: 1.155–1.383, *P* < 0.001), while LODS and SOFA were not correlated with the mortality (OR: 1.041, 95%: 0.992–1.092, *P* = 0.105; OR: 1.016, 95%: 0.981–1.052, *P* = 0.373) (Table 5).

**Table 5:** Logistic regression analysis of the four scoring systems for 30-day mortality among intensive care patients with sepsis.

Variable	Univariable		Multivariable	
	OR (95% CI)	*P*	OR (95% CI)	*P*
Age (Year)	1.027 (1.023–1.032)	< 0.001	1.015 (1.009–1.021)	< 0.001
Gender (Male)	0.900 (0.787–1.030)	0.125		
Septic shock	2.952 (2.493–3.496)	< 0.001	1.293 (1.050–1.593)	0.016
CAD	0.864 (0.733–1.017)	0.079	0.672 (0.557–0.810)	< 0.001
Diabetes	0.968 (0.833–1.124)	0.668		
Hypertension	1.174 (0.982–1.404)	0.078	0.809 (0.562–1.165)	0.255
CPD	1.265 (1.078–1.484)	0.004	1.219 (1.019–1.458)	0.031
Renal failure	1.263 (1.069–1.492)	0.006	1.024 (0.728–1.440)	0.891
LODS	1.351 (1.318–1.384)	< 0.001	1.041 (0.992–1.092)	0.105
SAPS II	1.078 (1.073–1.084)	< 0.001	1.061 (1.051–1.071)	< 0.001
SIRS	1.527 (1.409–1.655)	< 0.001	1.264 (1.155–1.383)	< 0.001
SOFA	1.241 (1.216–1.266)	< 0.001	1.016 (0.981–1.052)	0.373

*CAD* Coronary atherosclerotic heart disease, *CPD* Chronic pulmonary disease.

## Discussion

This study followed the latest definition of Sepsis-3 and selected four commonly used scoring systems to conduct a large-sample retrospective cohort study. Meanwhile, in the selection of patients, we strictly follow the standards of Johnson et al.^11,12^, for they are in charge of the MIMIC database, and some of them also work for the Beth Israel Deaconess Medical Center. In this way, the results we summarized could be more credible. By drawing ROC curves and comparing AUC, we found that the AUC of the four systems from large to small were as follows: SAPS II, LODS, SOFA, and SIRS, indicating that SAPS II has the best predictive value (SAPS II > 46.5 can predict the risk of 30-day mortality in intensive care patients with sepsis), followed by LODS, and the predictive value of SOFA and SIRS is relatively low. This ranking is almost consistent with the complexity ranking of the four scoring systems while the SAPS II is calculated from the worst value of 12 routine physiological measurements^5^. However, these items are easily available in the ICU, so the complexity of SAPS II may not affect its clinical application, even if clinicians are more inclined to use a concise and easily accessible scoring system to predict the risk of death.

As a diagnostic criterion for sepsis, SOFA has been shown to be effective in assessing the prognosis of patients with sepsis in large retrospective studies^3,14^, but the results of our study show that SOFA has no priority in predicting the mortality of intensive care patients. Therefore, it is necessary to adopt multiple scoring systems in the ICU management of sepsis. A previous study (n = 7932) showed that the predictive validity for in-hospital mortality of SOFA was not significantly different than the more complex LODS among ICU encounters with suspected infection, supporting the use of SOFA in clinical criteria for sepsis^15^. It is worth noting that the SIRS criteria, SOFA and LODS scores of the study were calculated for the time window from 48 h before to 24 h after the onset of infection, while the relevant scores calculated in our study were all derived from the first 24 h of admission. Although the starting time for the follow-up are not the same, our study also found that the results of LODS predicting the 30-day mortality of non-elderly patients are consistent with the above, however, in predicting the mortality of all sepsis patients, LODS has a slight advantage over SOFA. The finding seems meaningful. More than 60% of sepsis diagnoses are made in the elderly^16^, and it is therefore valuable to predict the 30-day mortality rate of the elderly, especially in ICU. For example, elderly patients not expected to survive sepsis may consider palliative care services to relieve pain and make death more peaceful for instance. However, compared to SAPS II, the predictive effectiveness of LODS and SOFA is much inferior. Our logistic regression analysis also found that LODS and SOFA were not correlated with 30-day mortality in intensive care patients with sepsis, but SAPS II was an independent risk factor. Therefore, SAPS II is undoubtedly a better scoring system choice for predicting the 30-day mortality of elderly or non-elderly patients with sepsis in ICU.

SIRS criteria in this study showed high sensitivity, its specificity and Youden’s index were very low. The criteria were once used for the diagnosis of sepsis 1.0^1^, but the SIRS syndrome is not only induced by infection, such as trauma, severe acute pancreatitis, and shock can all lead to SIRS, which may be the root cause of the lack of specificity of SIRS criteria. Previous studies have shown that SOFA, even qSOFA (for quick SOFA, using three criteria, assigning one point for SBP ≤ 100 mmHg, high respiratory rate ≥ 22 breaths per min, or Glasgow coma scale < 15), are superior to SIRS criteria in determining the ICU stay and mortality of patients^17,18^. It can be seen that the SIRS criteria are not appropriate for the diagnosis or the prognosis prediction of sepsis.

In the stratified analysis, the sensitivity and specificity of the different scoring systems markedly changed in the elderly and non-elderly, especially SIRS and SOFA. The SIRS includes only four items (temperature, heart rate, respiration, and white blood cells), which can easily lead to deviations in results. Moreover, previous studies have shown that SIRS is a prevalent feature of sepsis, should be an important component of the diagnostic process^19^, not of the prognostic process. Therefore, the difference between sensitivity and specificity of the stratified analysis also reflects that SIRS is not suitable for predicting 30-day mortality of sepsis. As for the SOFA, previous studies believe that regular and repeated scoring of SOFA can better understand the condition and disease development of the patients^20^. Then, the SOFA scoring performed on the first day of admission may not be sufficient to clarify the real status of the patients or predict the 30-day mortality, and may also potentially cause the difference between sensitivity and specificity of the stratified analysis. In addition, the sample size of elderly and non-elderly patients and the heterogeneity of the patients themselves are also potential factors leading to differences. We noticed that the sensitivity and specificity of the LODS and SAPS II in the stratified analysis are not much different, probably because the LODS provides an objective tool for assessing severity levels for organ dysfunction in the ICU^6^, while the SAPS II provides an estimate of the risk of death without having to specify a primary diagnosis^4^. The SAPS II always maintained the highest Youden’s index, which is even more confirmed its universality and robustness.

Traditional metrics of diagnostic performance such as sensitivity, specificity, and AUC only measure the diagnostic accuracy of one prediction model against another, but fail to account for the clinical utility. A model with a higher AUC is likely to be more valuable than one with a lower AUC but models with higher AUCs can sometimes lead to inferior outcomes^10^. DCA is a widely used method to measure the clinical utility of a specific model^21^, and can therefore inform the decision of whether to use a model at all or which of several models is optimal. DCA is graphically expressed as a curve with benefit score on the vertical axis and probability thresholds on the horizontal axis. A key concept of DCA is the “threshold probability”, where the expected benefit of treatment is equal to the expected benefit of avoiding treatment. The so-called “net benefit” is determined by calculating the difference between the expected benefit and the expected harm. One line is drawn to show what happens when no treatment is ever given (no net benefit. such as “the horizontal line” with ordinate of 0 in Fig. 4), and another curve is drawn as if all patients receive treatment irrespective of predicted results (such as “the diagonal line” in Fig. 4). For any given probability threshold, the curve with the highest benefit score at that threshold is the best choice^10^. If one curve is highest over the full range of probability thresholds, then the associated diagnostic approach would be the best decision for all patients^21^. In our study, regardless of whether it is for all included patients or stratified analysis (by age), the DCA curve of the SAPS II scoring system is the highest within the entire probability threshold range, indicating that using the SAPS II scoring system to judge the 30-day mortality of patients with sepsis and further deciding whether to conduct active intervention will yield the greatest benefits. The range under the DCA curve of the four scoring systems is almost the same as the corresponding AUC, again confirming the superiority of the SAPS II scoring system. It is worth mentioning that the DCA curve of the SIRS mostly overlaps with “the horizontal line” and “the diagonal line”, indicating that it is not suitable for clinical application.

We must acknowledge some limitations of our study: firstly, the ethnicities of the population included in this study are mainly white and black, and there may be ethnic differences. Therefore, the results may not be applicable to other ethnic groups, such as Asian or Hispanic; secondly, due to the diversity of the scoring system, this study selected just four existing representative ones, hoping to be easily applied to clinical decision-making. Even if SAPS II performs well, it may not be the best score for predicting the 30-day mortality of sepsis. Hou et al*.* built a model using machine learning technique by XGboost, and found that the net benefit for XGboost model was larger over the range of SAPS II score, which means the novel model is optimal and the SAPS II score inferior^22^. With the rapid development of artificial intelligence (machine learning) and medical big data, better predictive models may be developed in the future; thirdly, this study failed to distinguish patients with sepsis caused by different infection sites, and various scoring systems may have biased predictions of the prognosis. Once the infection sites of the MIMIC database are further supplemented, this issue will be solved. Last but not least, the Medcalc Statistical Software cannot be used for comparison after correction, the related significativity may be overestimated statistically in this study. Thus, prospective researches need to be conducted, and better predictors need to be further explored.

## Conclusions

Among the four commonly used scoring systems, the SAPS II score has the highest predictive value for 30-day mortality from sepsis, which is better than LODS, SIRS, and SOFA. The results of the DCA curves show that using the SAPS II score to predict the 30-day mortality of patients with sepsis to guide clinical applications may obtain the highest net benefit.
